# Would Antarctic Marine Benthos Survive Alien Species Invasions? What Chemical Ecology May Tell Us

**DOI:** 10.3390/md20090543

**Published:** 2022-08-24

**Authors:** Conxita Avila, Xavier Buñuel, Francesc Carmona, Albert Cotado, Oriol Sacristán-Soriano, Carlos Angulo-Preckler

**Affiliations:** 1 Department of Evolutionary Biology, Ecology, and Environmental Sciences, Faculty of Biology, University of Barcelona, 08028 Barcelona, Catalonia, Spain; 2 Biodiversity Research Institute (IrBIO), University of Barcelona, 08028 Barcelona, Catalonia, Spain; 3 Whitman Center, Marine Biological Laboratory, Woods Hole, MA 02543, USA; 4 Institut Català de Recerca de l’Aigua, c/Emili Grahit, 101 (Edifici H2O-ICRA), 17003 Girona, Catalonia, Spain; 5 Red Sea Research Center (RSRC) & Biological and Environmental Sciences and Engineering Division (BESE), King Abdullah University of Science and Technology, Thuwal 23955-6900, Saudi Arabia

**Keywords:** chemical defenses, polar biology, marine natural products, marine benthic macroinvertebrates, macropredation, micropredation, non-native alien species, invasive species, global change, crabs

## Abstract

Many Antarctic marine benthic macroinvertebrates are chemically protected against predation by marine natural products of different types. Antarctic potential predators mostly include sea stars (macropredators) and amphipod crustaceans (micropredators) living in the same areas (sympatric). Recently, alien species (allopatric) have been reported to reach the Antarctic coasts, while deep-water crabs are suggested to be more often present in shallower waters. We decided to investigate the effect of the chemical defenses of 29 representative Antarctic marine benthic macroinvertebrates from seven different phyla against predation by using non-native allopatric generalist predators as a proxy for potential alien species. The Antarctic species tested included 14 Porifera, two Cnidaria, two Annelida, one Nemertea, two Bryozooa, three Echinodermata, and five Chordata (Tunicata). Most of these Antarctic marine benthic macroinvertebrates were chemically protected against an allopatric generalist amphipod but not against an allopatric generalist crab from temperate waters. Therefore, both a possible recolonization of large crabs from deep waters or an invasion of non-native generalist crab species could potentially alter the fundamental nature of these communities forever since chemical defenses would not be effective against them. This, together with the increasing temperatures that elevate the probability of alien species surviving, is a huge threat to Antarctic marine benthos.

## 1. Introduction

Antarctic marine benthos comprise some of the oldest and most stable marine ecosystems in the world [1,2]. In these environments, marine communities consist of very diverse invertebrates, some sessile and suspensivorous, and other vagile and predators, building up a complex network of ecological interactions [3,4,5,6,7,8]. In these habitats, strong predation pressure and huge competition for resources exist [1,3,6,7,8,9,10], with interactions occurring there being crucial for structuring Antarctic communities [3,4,8]. Consequently, many effective defensive mechanisms have appeared along evolution to ensure species survival [11,12,13,14,15,16,17,18,19,20,21,22,23,24,25]. Defensive strategies in the Southern Ocean include the use of chemicals (marine natural products or MNPs) to obtain protection against potential predators, among many other roles [8,13,18,19,20,21,22,23,24,25,26]. Indeed, Antarctic macroorganisms do present a wide array of bioactive molecules that can be used in situ by the organisms but also may potentially be useful from a pharmacological perspective [23,24,26,27].

However, in Antarctica, the ecological role of marine natural products has only been analyzed in detail for some species or groups so far [8,13,19,20,21,22,28,29,30,31,32,33,34,35,36,37,38], and therefore, much remains to be investigated. Interestingly, it is well known that Antarctic benthic communities in shallow and shelf areas are dominated by epifaunal suspension feeders that are poorly adapted to resist shell-breaking (durophagous) predators [39,40], since they are absent in these ecosystems. The main macropredators here are sea stars and large nemerteans, along with micropredators, such as amphipod crustaceans [8,13,19]. Many invertebrate species, therefore, have been tested for chemical repellence against the widely distributed generalist omnivorous predator *Odontaster validus* [7,11,13,14,15,18,21,22,32,33,34,35,36,37,38,41,42,43,44], while other assays have been done against micropredator amphipods [7,8,13,16,18,19,21,25,33,34,35,41,42,43,44,45]. In general, repellent activity in Antarctic marine invertebrates has been reported to be comparable to that of temperate and tropical ecosystems [8,12,13,19,31,46,47,48]. This could suggest that perhaps their chemical defenses could be effective against opportunistic predators, whether sympatric or allopatric. In fact, Becerro and co-authors [49] concluded that chemical defenses from tropical sponges were as effective as those of temperate sponges using prey and predators from both latitudes. In addition, extracts from sponges from the Caribbean had similar repellent or palatable effects in predatory fish from the Caribbean and from the Red Sea, indicating that these are general responses by fish predators to sponge natural products, regardless of the geographic origin of the fish [50]. Antarctica, however, is peculiar due to its environmental isolation and stability. Thus, it would be possible that the Antarctic species developed specific chemical defenses against sympatric predators, which are useless against allopatric predators. This is particularly relevant if we consider that macropredators are mostly sea stars and nemerteans, as said above, with a scarce presence of fish or durophagous (shell-breaking) fauna [2,3,6,8,13,19,25,48,51,52].

The topic is also relevant in the context of global change, as Antarctica is suffering a dramatic increase in sea water temperatures [53,54]. In this context, any alien species arriving in Antarctic areas and finding an appropriate environment to survive could potentially become invasive and completely alter native communities. Indeed, Antarctica is not as isolated as once thought [55,56], and the arrival of non-native species to Antarctic shallow, slope, or shelf waters has recently been reported [57,58,59,60], deserving further monitoring. Actually, scientists are continuously warning about the problems produced by alien species worldwide since the potential effects in the native communities are not yet completely understood but could presumably be dramatic in many areas of the planet [60]. Among the potential species arriving now on Antarctic shores and shelves, different species of amphipods and crabs are found [57,58,59,61,62,63,64,65,66,67,68]. These animals are usually generalist predators able to feed upon many kinds of marine benthic invertebrates [69,70,71]. In fact, some king crabs are reported to feed on ophiuroids, gastropods and bivalve molluscs, echinoids, asteroids, holothurians, polychaetes, bryozoans, and poriferans, being omnivorous and opportunistic [72,73]. Then, the chemical defenses of the Antarctic benthic invertebrates would prove crucial to avoid being eaten by non-native amphipods and crabs.

As mentioned above, very few crabs currently inhabit Antarctic shallow waters [39,61,62,73,74,75]. In fact, the continental shelves in Antarctica are dominated by very rich communities of sessile macroinvertebrates and slow-moving epifaunal invertebrates that have evolved in the absence of durophagous predators for millions of years, at least since the last cooling event of Antarctica until ca. 16 Ma ago [63]. However, recently, two species of large crabs (Family Lithodidae) have been reported to be abundant at the slope, that is, *Paralomis birsteini* and *Neolithodes yaldwyni*, being present up to ca. 700 m depth [58,63,72,73,76]. There has been some discussion recently in the literature regarding whether these large crabs are recolonizing Antarctica from the deep due to the sea water temperature increase or whether they just remained unseen for decades and are now more often observed [39,40,58,61,62,63,66,67,68,72,73,74,75,76,77,78,79,80]. Regardless, their appearance could have dramatic negative effects on the Antarctic benthos, and this is starting to be of great concern [72,73,76]. No matter what their origin is (recolonizer, non-native, alien…), we find it very relevant to evaluate whether crabs would be repelled by the chemical defenses of Antarctic marine benthic invertebrates or not, especially if we consider that there are no other durophagous (shell crushing) predators in Antarctica that may control the growth and expansion of these new arrivals.

Because of this, we decided to analyze the feeding deterrence of several Antarctic species of marine benthic invertebrates against temperate potential generalist predators, namely amphipods and crabs. Our objectives were to (1) evaluate the ability of Antarctic marine benthic invertebrates to protect themselves by using marine natural products against potentially invasive or allopatric species (amphipods and crabs), and (2) compare the results with similar repellence assays previously performed against Antarctic sympatric predators (amphipods and sea stars). Our null hypothesis was that most chemical defenses found in their organic extracts would be equally effective against generalist macro- and micropredators, and therefore the benthic invertebrates tested would be protected against potentially invasive alien species. As we report below, this hypothesis is supported by our findings for amphipods but not for crabs.

## 2. Results

The extracted samples from Antarctic marine benthic macroinvertebrates yielded the amounts of lipophilic and hydrophilic fractions reported in Table 1. These fractions were prepared at natural concentrations to be tested against micro- and macropredators from the Mediterranean, providing different results, as follows.

### 2.1. Micropredation Experiments

**Figure 1:**
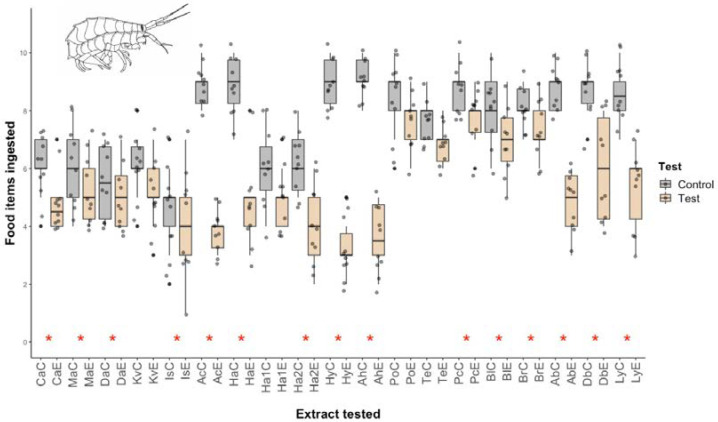
Micropredation results for Antarctic marine invertebrate lipophilic extracts against Mediterranean amphipods (Fam. Lysianassidae). *: statistically significant differences with respect to the control (*p* < 0.05) using the Wilcoxon test. Control boxes are shown in gray; extract lipophilic fractions in orange. Ca; *Clathria* sp. Ma; *Mycale acerata*. Da: *Dendrilla antarctica*. Kv; *Kirkpatrickia variolosa*. Is; *Isodictya* sp. Ac; *Axinella crinita*. Ha; *Haliclona* sp. Ha1; *Haliclona* sp1. Ha2; *Haliclona* sp2. Hy; *Hydroidea* sp. Ah; *Alcyonium haddoni*. Po; *Harmothoe* sp. Te; *Terebellidae* sp. Pc; *Parborlasia corrugatus*. Bl; *Bugula longissima*. Br; Cheilostomata sp. Ab; *Abatus* sp. Db; *Diplasterias brucei*. Ly; *Lysasterias* sp.

The results show that most of the lipophilic fractions from the Antarctic invertebrates tested were deterrent to the Mediterranean amphipods and that the differences from the controls are statistically significant (Figure 1). Only the two polychaeta species (*Harmothoe* sp. and the terebellid) and two sponge species (*Kirkpatrickia variolosa* and *Haliclona* sp. 1) showed no activity.

**Figure 2:**
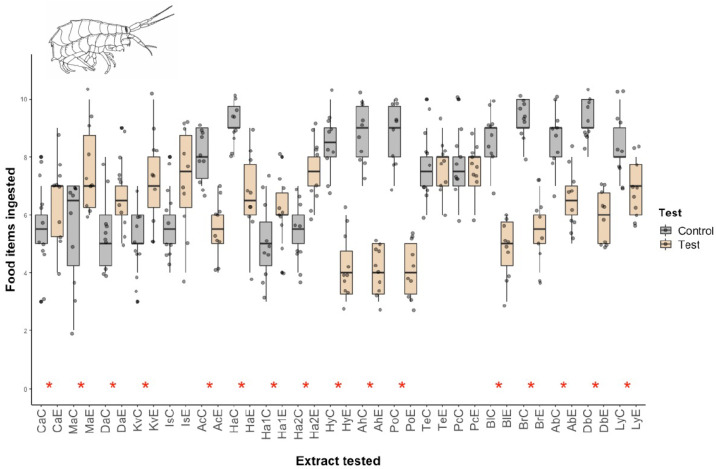
Micropredation results for Antarctic marine invertebrate hydrophilic extracts against Mediterranean amphipods (Fam. Lysianassidae). *: statistically significant differences with respect to the control (*p* < 0.05) using the Wilcoxon test. Control boxes are shown in gray; extract hydrophilic fractions in orange. Ca; *Clathria* sp. Ma; *Mycale acerata*. Da: *Dendrilla antarctica*. Kv; *Kirkpatrickia variolosa*. Is; *Isodictya* sp. Ac; *Axinella crinita*. Ha; *Haliclona* sp. Ha1; *Haliclona* sp1. Ha2; *Haliclona* sp2. Hy; *Hydroidea* sp. Ah; *Alcyonium haddoni*. Po; *Polynoidae* sp. Te; *Terebellidae* sp. Pc; *Parborlasia corrugatus*. Bl; *Bugula longissima*. Br; Cheilostomata sp. Ab; *Abatus* sp. Db; *Diplasterias brucei*. Ly; *Lysasterias* sp.

Similar results were obtained for the hydrophilic fractions, with most fractions displaying significant deterrence against Mediterranean amphipods (Figure 2). The only species showing palatability were the terebellid polychaete, the nemertean (*Parborlasia corrugatus*), and one sponge (*Isodyctia* sp.).

### 2.2. Macropredation Experiments

**Figure 3:**
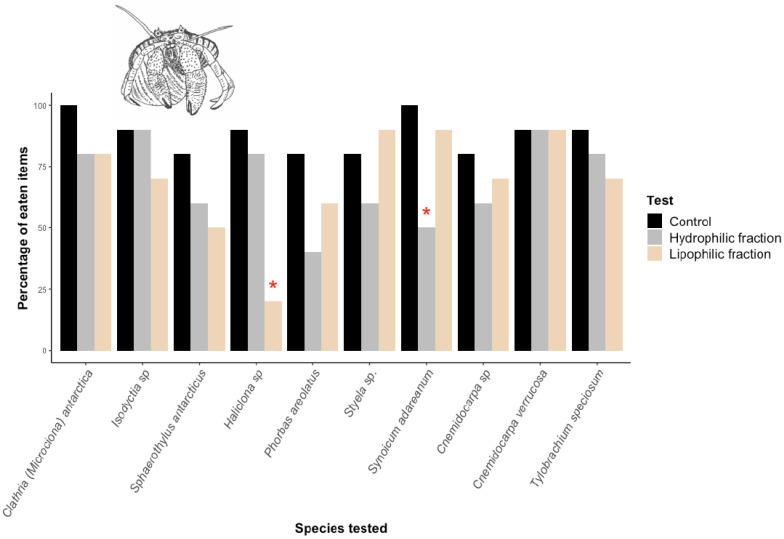
Macropredation results for Antarctic marine invertebrate extracts (sponges and tunicates) against the Mediterranean hermit crab *Dardanus arrosor*. *: statistically significant differences with respect to the control (*p* < 0.05) using the Exact Fisher test. Control results (%) are shown in black, lipophilic fractions in orange, and hydrophilic fractions in gray.

Our results show that most Antarctic species tested here are not chemically protected against predation by the generalist macropredator *Dardanus arrosor* (Figure 3). Only the sponge *Haliclona* sp 4 and the tunicate *Synoicum adareanum* showed deterrence in their lipophilic or hydrophilic fractions, respectively.

## 3. Discussion

To the best of our knowledge, this is the first study testing chemical extracts from Antarctic marine benthic invertebrates against potential predators from different geographical areas, and in particular, from the Mediterranean Sea. In cases where the repellent activity is similar to that reported against Antarctic predators, we may assume that the defensive mechanism works in an equivalent way in both ecosystems, thus being a broad chemical strategy that protects the Antarctic invertebrates against potential alien species (or similarly related allopatric species). This has also been reported in the literature at different latitudes [48,49]. Where the results are different, we may assume that the presence of these alien species (or other similarly related allopatric species) could potentially be a threat to the survival of the Antarctic invertebrates tested, as explained below.

### 3.1. Predation Experiments

Most Antarctic species tested here are chemically protected against Mediterranean amphipods (Fam. Lyssianassidae) but not against the Mediterranean hermit crab *Dardanus arrosor* (Table 2). These results indicate that Antarctic chemical defenses are crucial in benthic ecological interactions, as previously reported [7,8,11,13,14,15,16,18,19,20,21,22,25,26,30,32,33,34,35,41,42,43,44,45], but also that similar strategies may (or may not) work against predators from different environments depending on the predator tested. In our case, chemical defenses protect the marine invertebrates studied against micropredators (amphipods), which will not eat them, but not against macropredators (crabs), which may eat them (Table 2).

However, the number of species tested for some of the groups is still small (Table 1), and therefore, these data should be interpreted cautiously. Additionally, even if we used all the available samples for these assays, we could not use the same species in all the assays developed; therefore, more assays should be carried out to have a more complete picture of the repellent activities. Nonetheless, based on our results, we believe that deterrence against micropredators from a totally different environment (Mediterranean vs. Antarctic) seems to be based on the same defensive mechanisms, with the chemicals used having equivalent roles in the two ecosystems. The high diversity and abundance of amphipods in Antarctica, along with the absence of crabs, reinforce this assumption [6,8,25,39,40,61,70,81,82,83,84,85,86,87]. The extracts tested here may contain a variety of MNPs, including compounds such as alkaloids, terpenoids, polyketides, peptides, and others (see below). Further studies should evaluate the effects of the isolated molecules to further prove this common strategy against predators from different geographical areas.

Amphipods live in association with other macroorganisms, such as macroalgae, sponges, bryozoans, and other sessile macroinvertebrates [81,82,83,84,85,86,87]. Sessile macroinvertebrates may provide them with shelter and protection against potential predators by physical and chemical defense, as well as direct or indirect feeding resources [7,8,9,13,25]. Antarctic sessile macroinvertebrates have repellent effects against both Mediterranean (results reported in this study) and Antarctic amphipods (Table 2) [7,8,13,16,18,19,21,25,33,34,35,41,42,43], and this strategy of chemical defense may help them decrease the ecological pressure the amphipods may exert on them. However, other studies using different species indicate that the amphipod *Gondogeneia antarctica* prefers food with extracts of some Antarctic sponge species [86,88]. In this particular case, the amphipod does not seem to be responsible for the evolution of the chemical defenses in these sponges [86,88].

The potential negative effects of amphipods on sessile organisms consist not only in the small bites of an individual when trying to feed on them, but also in the effects of thousands of individuals trying to prey simultaneously upon them, as well as the clogging of the filtration systems, affecting feeding, respiration, and reproduction in the sessile macroinvertebrates [7,34,35]. Our results indicate that all tested species were chemically protected from a model species of allopatric amphipods from the Mediterranean Sea. It is of note that chemical deterrence was detected in lipophilic or hydrophilic fractions, and in some instances in both fractions. The single exception was the terebellid polychaete, which demonstrated a lack of deterrence for all tested fractions. The amphipod used for the assays is considered ecologically equivalent to the Antarctic species used in previous studies (*C. femoratus*, *G. antarctica*) [7,16,21,86,88]. Antarctic amphipods, as indicated above, are a rich and biodiverse group [81,82,83,84,85,86,87], and their speciose nature and high abundance are likely to have provided ample evolutionary pressure to cause sessile macroinvertebrates to evolve chemical defenses that have general effects against crustacean mesograzers. Thus, all these findings support the fact that chemical defenses are broadly effective and would protect the invertebrates tested here from putative alien amphipods arriving in Antarctic waters from temperate areas.

Regarding the hermit crab *Dardanus arrosor* (Herbst, 1796), it has been described as a generalist and opportunistic feeder as other similar related species [89,90,91]. Our study provides a new methodology for repellency experiments in the Mediterranean, being ecologically similar and comparable to the Antarctic experiments using the sea star *Odontaster validus* [7,11,13,14,15,18,21,22,32,33,34,35,41,42,43,44,45]. The hermit crab *D. arrosor* has proved here to be a good laboratory model for chemical ecology experiments, with good behavior and survival, as well as providing a rapid answer to experimentation assays. When comparing the results to those previously obtained by our group in Antarctica, we observe that some discrepancies may appear. This is the case with the sponge *Haliclona* sp. 4, which is repellent against the Mediterranean crab but not against the Antarctic sea star *O. validus*. Since we are comparing only a few species from different phyla, more studies are needed to ascertain why these different results occur and what the ecological meaning is. Instead, the tunicate *S. adareanum* is repellent against both macropredators [18]. Remarkably, however, most invertebrate species tested against the Mediterranean hermit crab *D. arrosor* were not repellent in our assays (Table 2).

Chemical defenses against macropredators would therefore have a very narrow effect, only against Antarctic macropredators (*O. validus*) so far [7,11,13,14,15,18,21,22,33,34,35,41,42,43]. This could perhaps be related to the environmental stability and relative isolation of these Antarctic ecosystems [1,2], which could have driven a very specific mechanism of chemical defense against specific Antarctic macropredators. The absence of a wide diversity of potential crab macropredators in Antarctic benthic communities [9,39,40,61,62,73,74,75] may also have contributed to this fact, in contrast to the presence of a wider range of micropredators (amphipods), as reported above. This means that these macroinvertebrates would not be chemically protected if this crab or a similar temperate alien species reached Antarctic waters. Both the non-native crabs (*Halicarcinus planatus* and *Carcinus maenas*) and the large crabs from deeper waters found so far in Antarctica (*Paralomis birsteini* and *Neolithodes yaldwyni*) are generalist predators that could potentially feed on all these benthic macroinvertebrates [58,59,66,72,73,92]. The effects of a generalist crab on shallow-water Antarctic benthic communities could therefore be tremendous if an alien species like this one arrives and settles in Antarctica.

It is now well established that decapods largely became extinct millions of years ago on the shelf and slope of Antarctica, and that is only recently that it has been discovered that several species of king crabs are positioned to recolonize Antarctic waters [58,63,66,68,72,73,76,77]. The long-considered rationale for their exclusion was the known incapacity of decapods to regulate magnesium ions in their hemolymph at low temperatures [61,62,66,75,77,78,79]. With the warming of the Antarctic circumpolar current, this physiological barrier is likely lifted, allowing crabs to move up the slope toward the shelf [58,66,73].

In addition to king crabs moving up the slope from deep water, several small species of crabs, *Halicarcinus planatus* and *Carcinus maenas,* have already been detected in shallow coastal waters of Antarctica [58,59,66]. These classic invasive species have been found in very low numbers to date but do pose a risk for future colonization [93,94,95]. Similar to king crabs, these smaller crabs are generalist predators and could contribute to dramatic and devastating impacts on unique and fragile Antarctic benthos.

### 3.2. Marine Natural Products

Most repellent marine natural products are lipophilic [27,96], but many bioactive compounds have different polarities [8,24,25,27,97,98,99]. For this reason, we used both the lipophilic and hydrophilic fractions from the macroinvertebrates tested and found, in fact, some differences, as reported below.

In Porifera, a group particularly rich in MNPs, *Clathria* sp. (Calcarea class), showed deterrence against amphipods in both extract fractions. There are very few studies on the chemistry of calcareous sponges, but it is known that some may possess antifouling compounds in their extracts (*Leucetta leptorhapsis* and *L. antarctica*; Fam. Chlatrinidae) [12,100,101]. As far as we know, the compounds have not yet been identified, and it is unknown whether those or similar compounds are responsible for the deterrent activity reported here.

**Figure 4:**
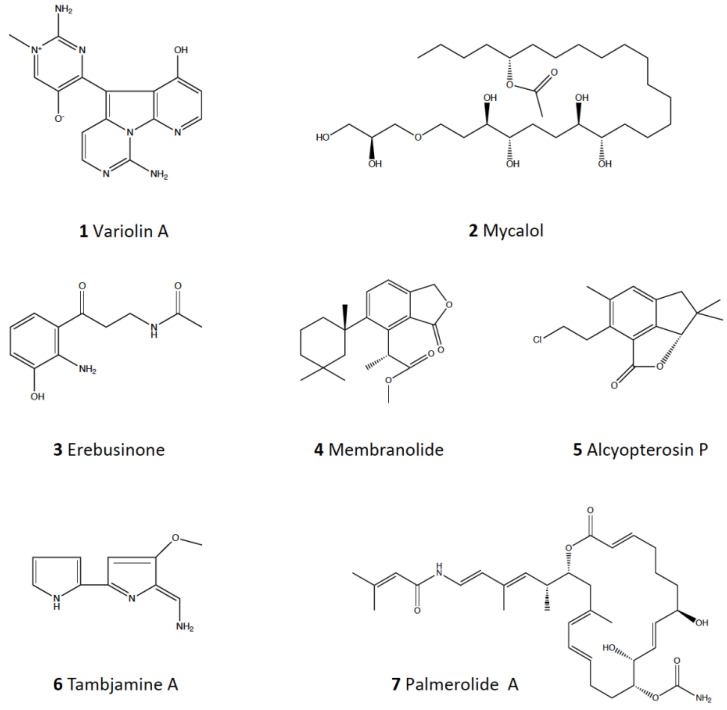
Chemical structures of marine natural products from some Antarctic macroinvertebrates. **1** Variolin A^20,101–104^; **2** Mycalol^109^; **3** Erebusinone^111–114^; **4** Membranolide^115–123^; **5** Alcyopterosin P^42,126–129^; **6** Tambjamine A^20,146^; and **7** Palmerolide A^150^.

*Kirkpatrickia variolosa*, instead, is a demosponge with well-known chemistry [20,26,101,102,103,104]. The alkaloid variolins (**1**) (Figure 4) have been described as relevant bioactive molecules [8,26,101,102,103,104] and could probably be the responsible molecules for the described repellence found here and in previous assays using *Odontaster validus*. Remarkably, only the hydrophilic fraction was repellent here, where organohalogens are probably found. Further assays with the isolated compounds from *K. variolosa* should more precisely establish the molecule responsible for feeding repellence.

*Axinella crinita* showed repellence in both fractions tested against amphipods, but no information has been available on its chemistry thus far. However, *Axinella* species in other geographic areas are characterized by the presence of alkaloids, peptides, and terpenoids, with 234 studies published on them so far [105].

Similarly, *Haliclona* species possess repellent activity against amphipods in both fractions tested here. These results agree with the previously reported activity of extracts of these sponges against the Antarctic amphipod *Cheirimedon femoratus* [16,33]. Regarding the chemistry of the *Haliclona* species, some studies have described antifouling activity in both lipophilic and hydrophilic fractions in *Haliclona dancoi,* as well as other bioactivities of its compounds [106,107]. These previously reported activities could be related to the deterrence found in our samples, although some variability may exist in the different species tested, some of which have not been identified to the species level so far. In fact, *Haliclona* is a chemically very rich sponge genus all over the world, containing alkaloids, quinones, terpenoids, polyacetylenes, peptides, lactones, and other compounds, as reported in 413 studies published until today [108].

The results obtained for the demosponge *Mycale acerata* are in agreement with the literature, being both fractions deterrent against Mediterranean amphipods, as for the Antarctic ones [12,13,14]. The main chemical behind this activity could be mycalol (**2**), a bioactive polyoxygenated glyceryl alkyl ether (Figure 4) [109], although assays with the isolated compound should be performed to demonstrate this. *Mycale* is also a chemically rich sponge genus, with 219 compounds cited worldwide as of today [12,13,14,110].

The demosponge *Isodyctia* sp. also presented deterrence in the lipophilic fraction, in agreement with previously reported repellent activity in *Isodictya spinifera* and other *Isodyctia* species tested previously [11,16,33,45,111]. Antarctic *Isodictya* species (*I. antarctica, I. erinacea, I. setifera*) have been studied over the years for their chemistry [112,113,114], with some of these described chemicals perhaps related to the repellence found here in our assays, such as the alkaloid eribusinone (**3**) (Figure 4). Again, tests with the isolated compound should be conducted to ascertain this.

Finally, *Dendrilla antarctica* also presented deterrence in our assays against amphipods in both hydrophilic and lipophilic fractions. Their natural products have been described as diverse and bioactive [115,116,117]. Similarly, the related *D. membranosa* (now assigned to *Dictyodendrilla pallasi*) was described as presenting antifouling activity among a wide array of bioactivities [12,30,106,118,119,120,121,122,123,124,125]. The natural products in these species include a variety of molecules that could perhaps be involved in the deterrence described here, such as many diterpenoids. An example of a *Dendrilla* compound is membranolide (**4**) (Figure 4).

Antarctic Cnidaria are also very rich in chemical compounds, particularly the genus *Alcyonium* [8,13,16,19,20,21,24,27,42,126,127,128,129]. *Alcyonium haddoni* has been previously evaluated for repellence against the Antarctic amphipod *C. femoratus,* and its lipophilic fraction has shown repellence [16,42]. Our results agree with this. The chemicals behind this repellence could be illudalanes, such as alcyopterosin P (**5**) (Figure 4) [42,129]. Similarly, the hydroids tested here were also repellent, as the hydroids from other latitudes which possess steroids and some monoterpenes [130,131,132,133,134,135,136,137]. The particular repellent compounds remain to be further identified.

The Nemertean *Parborlasia corrugatus* is a large worm-shaped organism reported to be a relevant generalist predator [138,139,140]. *P. corrugatus* is chemically protected against some Antarctic fish, and it is toxic to the sperm of the Antarctic sea urchin *Sterechinus neumayeri* [138,139,140]. *P. corrugatus* segregates copious amounts of acidic mucous secretions (pH = 3.5) reported to be toxic [140] and containing the cytotoxic neuropeptide parbolysine [138,139,140]. Our results agree with all that, as well as with the fact that its lipophilic fraction is repellent against the Antarctic sea star *O. validus* [45].

The Annelid polychaetes tested here, a terebellid and a polynoid (*Harmothoe* sp.), are living in close association with *Dendrilla antarctica* and *Mycale acerata* sponges (unpublished data from the authors). For the terebellid, the protection provided from the sponges may be effective enough against predators so that the worms do not need chemical defenses to be protected. These worms also possess a tube that provides physical protection. This is also in agreement with our previous assays showing no repellent activity in the terebellid *Pista spinifera* against the sea star *O. validus* [45]. In contrast, Polynoids are vagile animals that present protective scales. *Harmothoe* sp. is repellent to amphipods in their hydrophilic fraction. No information has been available so far regarding their chemistry. In other latitudes, polychaetes are known to present mostly peptides and some heterocyclic compounds, as well as an alkaloid, with 25 studies reported to date [141].

Antarctic Bryozoa present a rich array of MNPs [13,20,34,35,41,142,143,144], thus they can protect themselves from potential predators by chemical compounds against putative micropredators, such as amphipods. Since all the bryozoans tested here were repellent against Mediterranean amphipods, we assume that their chemical defenses have a broad spectrum. These results also agree with our previous results in assays with the Antarctic amphipod *Cheirimedon femoratus*, where most bryozoans displayed chemical repellence [16,34,35,41]. Both *Bugula longissima* and the unidentified species showed repellence in the hydrophilic extracts, in agreement with previous studies against Antarctic amphipods [16,34,35,37,41]. *B. longissima* has been reported to present bioactive tambjamine A (**6**) (Figure 4). Tambjamines are very active alkaloids that could potentially be responsible for this activity [20,145,146]. Further studies should test the isolated tambjamines to further prove this effect. In contrast, the related species *Bugula dentata* was not repellent against Antarctic amphipods in previous assays, and it is hypothesized that in that case mechanical defenses may have a preponderant role [34,35].

For Echinoderms, we tested only vagile species, one sea urchin, and two sea stars, which in general are less chemically protected than sessile invertebrates in Antarctica [13,22,23,25,45]. However, even if many of these vagile species may actively (often slowly) escape from predators, many also possess chemical defenses [13,22,23,25,45]. Both the sea stars *Diplasterias brucei* and *Lisasterias* sp. and the sea urchin *Abatus* sp. are repellent to Mediterranean amphipods. *D. brucei* has been reported to contain asterosaponins and steroids [147,148]. No information is available regarding the MNPs that could be present in the *Lisasterias* and *Abatus* species thus far, although some bioactive compounds have been reported in other Antarctic starfish [25,26,27].

Antarctic Tunicates are also a chemically rich group, with many bioactivities described [8,13,15,18,19,20,21,24,25,26,45,88,149,150,151,152,153,154,155,156,157]. Here, however, the species tested were not repellent in the assays against crabs, except for the hydrophilic extract of the colonial *Synoicum adareanum*. This contrasts with previous results in assays with Antarctic predators, where they were repellent [8,13,15,16,18,19,20,21,24,25,26,45,88]. *S. adareanum* possesses many chemicals, such as polyketides palmerolides (**7**) (Figure 4) [150,151,154,155]. The hydrophilic fraction may also contain these or perhaps other compounds that could produce this repellence. *Styela* and *Cnemidocarpa* species are individual tunicates, very abundant, and with rapid growth, which may contribute to their lack of unpalatability, even though *Cnemidocarpa* has some known chemistry [30]. Other Antarctic tunicate species have previously been reported to possess repellent compounds against the sympatric macropredator *O. validus* [15,16,18].

### 3.3. Climate Change and Alien Species

Temperatures are increasing in Antarctic waters [53,54]. These have many potential effects at physiological and ecological levels that may affect Antarctic benthic macroinvertebrates and force changes in their biodiversity [158,159,160,161]. The arrival of alien species that may settle and survive in Antarctic waters due to the warmer climate represents a dramatic threat to these ecosystems [57,59]. Within potential non-native species, amphipods, and crabs have been reported [57,58,59,61,62,63,64,65,66,67,68,72,73,74,76,77,78,79,80]. These non-native species may arrive transported by ballast water or also on macroalgal rafts, and could potentially survive in particularly warm areas, such as the volcano caldera of Deception Island [57,59]. Our data demonstrate that Antarctic benthic macroinvertebrates are likely to be chemically protected against equivalent amphipod micropredators from temperate waters (i.e., Mediterranean). However, they are less likely to be chemically protected against ecologically equivalent temperate crabs (i.e., Mediterranean). These results indicate that non-native crab species may potentially decimate Antarctic marine benthic invertebrate communities if they move into or are introduced to shallower areas and are favored by climate change. This has already been described as occurring in other geographic areas of the planet, where invasive king crabs have eliminated ca. 15% of the Arctic coastal population of sea urchins *Strongylocentrotus* spp., and a reduction in both benthic biodiversity and biomass due to king crabs was described in the Barents Sea [69,93,94,95,162,163,164,165]. All of these would sum up to the already existing effects of rising temperatures on the biodiversity and distribution of the Antarctic marine benthos. Further studies should be performed to better understand these mechanisms and how these ecosystems can be protected, if this is still possible, against the potential invasion of non-native alien crabs in the Anthropocene era.

## 4. Materials and Methods

### 4.1. Sample Collection and Extraction of Antarctic Macroinvertebrates

The available samples in our laboratory at UB were used for the assays (Table 1). Most samples were collected by scuba-diving during the ACTIQUIM projects at Deception Island (South Shetland Islands, Antarctica) in 2013 at 15–22 m depth (exceptions are detailed in Table 1). The water temperature in the area ranges between −1 °C and 4 °C (data from the authors). Species were taxonomically identified previously during the mentioned projects to the lowest possible rank. Abundant representative species from different phyla were selected for the different assays, considering the amount of material available (Table 1). All these samples were kept in the freezer at −20 °C until used. Voucher specimens were kept frozen when enough material was available at our lab (BEECA dept., UB, Barcelona, Catalonia, Spain).

Detailed results on the yields obtained from the extractions are shown in Table 1. Extractions were carried out as usual in the previously described protocols [16,33,34,35,44,45,166,167]. Briefly, acetone was used to extract the natural products at room temperature and using ultrasounds, evaporated, and then sequentially fractionated into a lipophilic fraction (diethyl ether fraction) and a hydrophilic fraction (butanol fraction). Water residues were not used here. All fractions were dried under the rotavap, and wet and dry weights were obtained to calculate the natural concentrations based on mass for each species tested. Samples were prepared to obtain the desired concentrations for the assays in each case.

### 4.2. Micropredation Experiments

Amphipods of the *Lysianassidae* Family (Decapoda: Amphipoda) were collected in three localities of the Catalan Coast, namely Blanes (La Selva), El Masnou (Maresme), and Montgat (Maresme). A few thousands of organisms were captured by hand using a mesh and a plastic bag between 0–2 m depth in May 2016. The most abundant ones, the lysianassids, were separated under a microscope to obtain enough specimens for the assays. Lysianassid amphipods are ubiquitous and eurybathic organisms with a wide variety of feeding strategies but mostly opportunistic predators [81,82,85]. The captured amphipods were kept in 30 L of oxygenated aquaria with fresh sea water at 16 °C at the UB for acclimatation. Water was changed daily. After five days of starvation, the amphipods were used in the assays.

Artificial food pearls were prepared as previously reported [16,33,42]. The pearls contained only the solvent (controls) or the fraction tested (lipophilic or hydrophilic) at the natural concentration. Our method is based on the Spherification Kit by the cook Ferran Adrià [168]. Briefly, 0.8 g of Phytoplan food is mixed with the solvent (containing or not the fraction to test), some colorant, and a solution of 0.05 g alginate in 10 mL distilled water. This mixture is dropped, forming pearls (3 mm diameter) in a solution of CaCl_2_. After 5 min in the solution, the pearls are collected and used for the assays.

Assays were carried out using 10 replicates for each assay. Amphipods were used only once in the assays. Each assay consisted of 10 1 L-bottles containing 15 amphipods, 10 control pearls, and 10 treatment pearls in fresh sea water. After 4 h, the number of pearls was counted, and notes were taken on whether they were completely eaten or not.

To observe whether differences exist in the treatments with respect to the controls, a Wilcoxon test was used [16,33,42]. The confidence index was 95%, and the software used was SPSS. Graphic visualization was performed in R version 4.2.0 with the *ggplot2* package [169,170].

### 4.3. Macropredation Experiments

The hermit crab *Dardanus arrosor* (Herbst, 1796) (Decapoda: Anomura) was selected for the assays because it is a common pagurid that is easy to collect in our area. Pagurids have been successfully used in chemical ecology experiments in other geographic areas [90,91,171]. Hermit crabs are common generalist omnivorous species that use different feeding strategies and include opportunistic habits [89,91,171].

Around 80 specimens of *D. arrosor* were collected off the Blanes harbor (La Selva, Girona, Catalonia, Spain) by two fishing boats, the “Estelada” and “La Milagros”, between April and June 2016. The collection depth was between 50 and 80 m. The size of the shells was between 8 and 11 cm long. Animals were transported to the laboratory at the UB and kept in 50 L aquaria with running filtered sea water at 14 °C for acclimatation. They were fed small shrimp pieces every three days. Before the assays, the crabs were in starvation for 3 days.

Assays were performed using the same methodology usually employed with the Antarctic sea star *Odontaster validus* [11,15,18,21,33,34,41,42,44,45]. Natural concentrations of the extract fractions were incorporated into shrimp cubes (0.5 cm^3^; 13.09 +/− 3.43 mg dry weight). A total of 10 shrimp pieces were prepared as replicates for each assay. Shrimp cubes were coated and left to dry under the hood for an hour before the assays. Each crab was placed in a 2.5 L container with fresh sea water and offered one piece of shrimp. Thus, each assay consisted of 10 containers with crabs offered control shrimp pieces (treated only with the solvent) and 10 containers with crabs offered treatment shrimp pieces. After 2 h, the assay was finished, and the number of shrimp pieces eaten was counted. Previous trials in our lab showed that 2 h was enough for a significant assay.

The results were analyzed using the Fisher Exact test [172]. The software used was SPSS, while the *ggplot2* package in R version 4.2.0 was used for graphics [169,170].

## 5. Conclusions

The absence of a broad suite of chemical deterrents from a variety of Antarctic marine invertebrates to deter feeding in a model species of crab should be of great concern. This is because crabs may soon recolonize the Antarctic shelf as climate change warms the Antarctic, either by migrating up from the deep sea surrounding the continent or through the establishment of invasive species. This lack of chemical defenses combined with a pattern of weak calcification would make Antarctic benthic organisms and their communities highly vulnerable. In contrast to crabs, the present study indicates that allopatric amphipods respond broadly to a wide suite of Antarctic marine invertebrate chemical feeding deterrents, and therefore should new amphipod species arrive in the warming waters of Antarctica, they are less likely to have an impact on the ecology of Antarctic benthic marine communities.

## Figures and Tables

**Table 1:** Species selected and yields obtained from the extractions of the Antarctic marine invertebrates used in the experiments. Except otherwise indicated, samples were collected at Deception Island, South Shetland Islands, Antarctica (S62°59′32.7″; W60°33′46.6″) by SCUBA diving at 15–22 m depth, during the ACTIQUIM 4 cruise (2012–2013).

Species	Wet Weight (g)	Dry Weight (g)	Liphophilic Extract (g)	Hydrophilic Extract (g)
PORIFERA				
*Clathria (Microciona) antarctica* (Topsent, 1916) ^1^	32.1	9.15	0.07	0.15
*Clathria* sp.	38.39	5.18	0.43	0.06
*Mycale (Oxymycale) acerata* Kirkpatrick, 1907	17.37	2.73	0.47	0.03
*Dendrilla antarctica* Topsent, 1905	35.52	1.34	0.24	0.10
*Kirkpatrickia variolosa* (Kirkpatrick, 1907)	67.41	15.34	0.48	0.29
*Isodyctia* sp. 1 *	10.56	1.26	0.45	0.08
*Isodyctia* sp. 2 *	41.13	5.42	0.28	0.12
*Axinella crinita* Thiele, 1905	85.69	12.14	0.04	0.03
*Sphaerothylus antarcticus* Kirkpatrick, 1907	53.43	12.47	0.22	0.04
*Haliclona* sp. 1 *	45.19	2.59	0.52	0.22
*Haliclona* sp. 2 *	19.54	1.30	0.28	0.16
*Haliclona* sp. 3 *	76.32	7.06	0.25	0.21
*Haliclona* sp. 4 *	124.76	8.43	0.46	0.15
*Phorbas areolatus* (Thiele, 1905) ^1^	103.18	23.79	0.32	0.15
CNIDARIA				
Hydroidea sp. ^2^	30.15	1.53	0.03	0.04
*Alcyonium haddoni* Wright & Studer, 1889	17.01	1.09	0.01	0.05
ANNELIDA				
*Harmothoe* sp.	1.42	1.14	0.05	0.01
Terebellidae sp.	64.5	26.25	0.43	0.02
NEMERTEA				
*Parborlasia corrugatus* (McIntosh, 1876)	66.83	6.41	0.03	0.09
BRYOZOA				
*Bugula longissima* Busk, 1884	21.96	1.37	0.06	0.06
Cheilostomata sp.	44.85	3.88	0.15	0.07
ECHINODERMATA				
*Abatus* sp.	21.57	6.27	0.05	0.04
*Diplasterias brucei* (Koehler, 1907) ^3^	17.02	5.02	0.17	0.10
*Lysasterias* sp.	68.92	15.10	0.64	0.48
TUNICATA				
*Styela* sp.	84.91	1.63	0.07	0.08
*Cnemidocarpa* sp.	66.21	2.35	0.05	0.13
*Cnemidocarpa verrucosa* (Lesson, 1830)	71.52	2.46	0.07	0.12
*Synoicum adareanum* (Herdman, 1902)	78.53	2.67	0.04	0.19
*Tylobranchion speciosum* Herdman, 1886	4.45	0.11	0.01	0.02

^1^ Collected at O’Higgins station, Antarctic Peninsula (S63°19′30.6″; W57°57′08.6″); ^2^ Collected at Schmidt Peninsula (S63°22′43.4″; W58°4′55.1″). ^3^ Collected at Barrios Island, Trinity Peninsula (S63°17′24.7″; W58°43′33.5″). * Voucher specimen(s) are kept at our sample collection at the BEECA department (UB).

**Table 2:** Summary of activities against Mediterranean and Antarctic macro- and micropredators by phylum. Data include samples tested in this study, as well as in previous works, and are shown in percentages. For data from different studies, the mean percentage and standard deviation were calculated. nt: not tested.

Group/Activity (%) against:	Mediterranean Macropredators (*D. arrosor*) 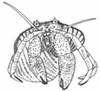	Antarctic Macropredators (*O. validus*) 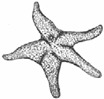	Mediterranean Micropredators (Amphipoda: Lyssianasidae) 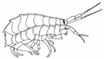	Antarctic Micropredators (*C. femoratus*) 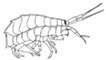
Porifera	20 ^1^	55.2 ± 26.9 ^11,21,33,44,45^	100 ^1^	100 ± 0 ^16,21,33^
Cnidaria	nt	80 ± 19.4 ^11,21,42,45^	100 ^1^	100 ± 0 ^16,21,42^
Annelida	nt	25 ± 35 ^11,45^	50 ^1^	nt
Nemertea	nt	50 ± 70.7 ^11,45^	100 ^1^	nt
Bryozoa	nt	49.7 ± 46.6 ^11,21,34,41,45^	100 ^1^	50 ± 57.7 ^16,21,34,41^
Echinodermata	nt	62.5 ± 31.1 ^11,21,45^	100 ^1^	0 ± 0 ^16,21^
Tunicata	20 ^1^	93.3 ± 14.9 ^11,15,18,21,45^	nt	100 ± 0 ^16,18,21^

^1^ This study; ^x^ rest of references as in the list.

## Data Availability

Not applicable here.
